# Microfluidic anodization of aluminum films for the fabrication of nanoporous lipid bilayer support structures

**DOI:** 10.3762/bjnano.2.12

**Published:** 2011-02-11

**Authors:** Jaydeep Bhattacharya, Alexandre Kisner, Andreas Offenhäusser, Bernhard Wolfrum

**Affiliations:** 1 Peter Grünberg Institute, PGI-8/ICS-8, Forschungszentrum Jülich GmbH, Leo-Brandt-Str., 52425 Jülich, Germany and Jülich - Aachen Research Alliance (JARA - FIT), Germany

**Keywords:** anodization, lipid bilayer, microfluidics, nanofabrication, nanoporous alumina

## Abstract

Solid state nanoporous membranes show great potential as support structures for biointerfaces. In this paper, we present a technique for fabricating nanoporous alumina membranes under constant-flow conditions in a microfluidic environment. This approach allows the direct integration of the fabrication process into a microfluidic setup for performing biological experiments without the need to transfer the brittle nanoporous material. We demonstrate this technique by using the same microfluidic system for membrane fabrication and subsequent liposome fusion onto the nanoporous support structure. The resulting bilayer formation is monitored by impedance spectroscopy across the nanoporous alumina membrane in real-time. Our approach offers a simple and efficient methodology to investigate the activity of transmembrane proteins or ion diffusion across membrane bilayers.

## Introduction

In recent years nanoporous alumina membranes have gained increased attention for technical and biological applications due to their versatile implementation as biointerfaces and ease of fabrication [1–8]. Their applications range from serving as template structures in nanofabrication technology [9–20] to their direct use as functional interfaces for controlled release of molecules [21–23], co-culture development [24], or biosensing [25]. For example, Steinem et al. have demonstrated the advantages of using nanoporous membranes as support structures for lipid bilayers, which allows the monitoring of the activity of membrane proteins [26–30]. Although solid lipid support structures [31] may exhibit versatile functionality for lab-on-a-chip applications [32], only porous substrates can address the backside of the membrane via a direct fluidic interface. The advantage of nanoporous substrates over other membrane spanning systems using microapertures lies in the increased stability caused by the small pore size [33–36]. Nevertheless, alumina membranes themselves are quite brittle and therefore easy to break if mechanical handling is required. Here, we present an approach of directly fabricating alumina membranes in a microfluidic environment which allows the monitoring and manipulation of membrane characteristics during the fabrication process. We demonstrate the formation of lipid bilayers on top of the nanoporous membrane which is monitored using impedance spectroscopy.

## Experimental

**Figure 1:**
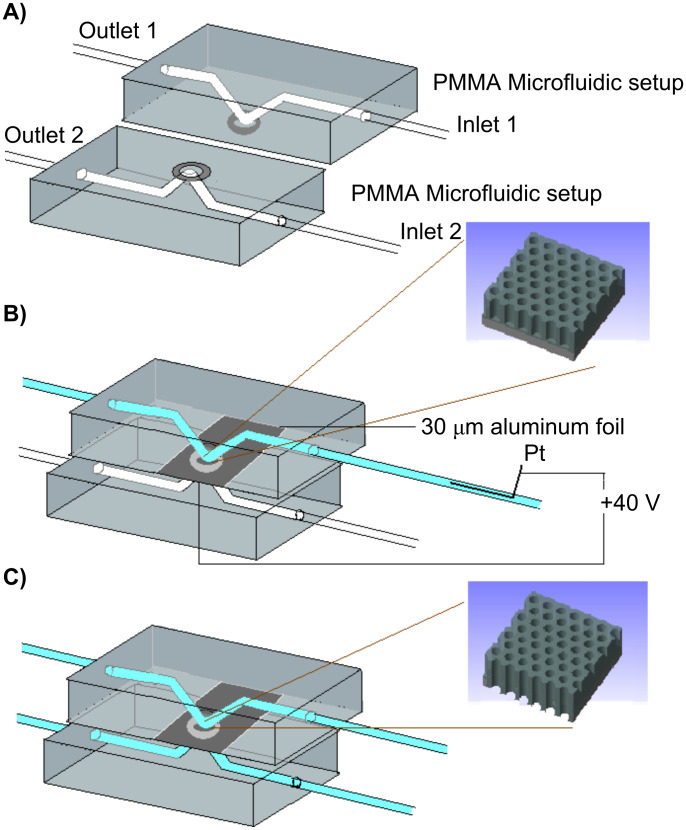
A) Schematics of the flow cell design for microfluidic anodization. B) An aluminum substrate is anodized at 40 V under a constant flow of oxalic acid inside a microfluidic cell to form a nanoporous alumina membrane. C) After the anodization is complete, the remaining alumina at the bottom of the pores is opened by injecting phosphoric acid into both fluidic channels.

The experimental setup for the microfluidic anodization approach is shown schematically in Figure 1.

The aluminum substrate, either a 30 µm thick aluminum foil or a thin aluminum film on an aperture released silicon support structure [21], was inserted into a PMMA dual-line flow cell. Oxalic acid (0.3 M) was injected into one of the inlets via pressure controlled flow (Fluigent, MFCS 4C, France) and contacted the exposed aluminum substrate in a localized area of 0.2 mm^2^. The aluminum was then anodized under constant voltage conditions. Thus, 40 V were applied between the aluminum and a platinum counter electrode, which was inserted in the flow cell, approximately 2 cm upstream of the substrate. The aluminum anode was directly contacted outside of the flow cell. Completion of the anodization was indicated by a steep drop in the anodization current indicating the formation of a residual alumina barrier layer. Oxalic acid was then rinsed from the channel and 5% phosphoric acid injected into the upper and lower channels to remove the remaining alumina film at the bottom of the nanoporous membrane. The dissolution of the barrier layer was monitored via impedance spectroscopy (10 Hz to 10 kHz) across the nanoporous alumina membrane by a modular electrochemical system [Autolab (PGSTAT 100/FRA2), Eco Chemie Utrecht, The Netherlands] using silver/silver chloride electrodes inside the fluidic channels on both sides of the membrane. A decrease in the recorded impedance indicated opening of the pores. Some of the nanoporous films were removed from the flow cell and investigated by scanning electron microscopy (Gemini 1550 VP, Carl Zeiss, Jena, Germany).

To prepare the nanoporous membrane for lipid bilayer formation, the nanoporous alumina surface was first subjected to silanization. The silanization was carried out in the solution phase according to the method described by Steinle and coworkers [37–38] with slight modifications. Briefly, a 10% (v/v) solution of (3-aminopropyl)triethoxysilane (APTES) was prepared in pure ethanol. The solution was mixed with a 0.1 M acetate buffer (pH 5.1) to a final concentration of 5% (v/v) buffered APTES. This solution was stirred mechanically for 5 min and passed through the microfluidic channel at 500 µL/h for 2 h at 22 °C. The whole system was then cured at 60 °C for 60 min.

The lipid bilayer on the modified nanoporous alumina surface was prepared by the method of liposomal fusion [39]. The liposomes were prepared from 1-palmitoyl-2-oleoyl-*sn*-glycero-3-phosphocholine (POPC, Avanti Polar Lipids, U.S.A.) by the following method. First, 5 mL of a lipid chloroform solution (5 mg/mL) were vacuum dried in a glass vessel. Then, a phosphate buffered saline (5 mL, 0.9% NaCl, 100 mM phosphate buffer, pH 7.2) was added to form multilamelar vesicles. Sonication and extrusion (Avanti Polar Lipids, U.S.A.) were performed to produce unilamellar small vesicles of approximate sizes between 60 and 80 nm as determined by dynamic light scattering (Dynapro, Wyatt Technology Corporation, U.S.A.). The vesicle solution was then injected into the microfluidic channel for the synthesis of the lipid bilayer on the modified alumina membrane. The formation of the lipid bilayer on the nanoporous support structure was monitored by impedance spectroscopy in the range of 10 Hz to 10 kHz with a root mean square amplitude of 10 mV as measured by the Autolab system.

## Results and Discussion

**Figure 2:**
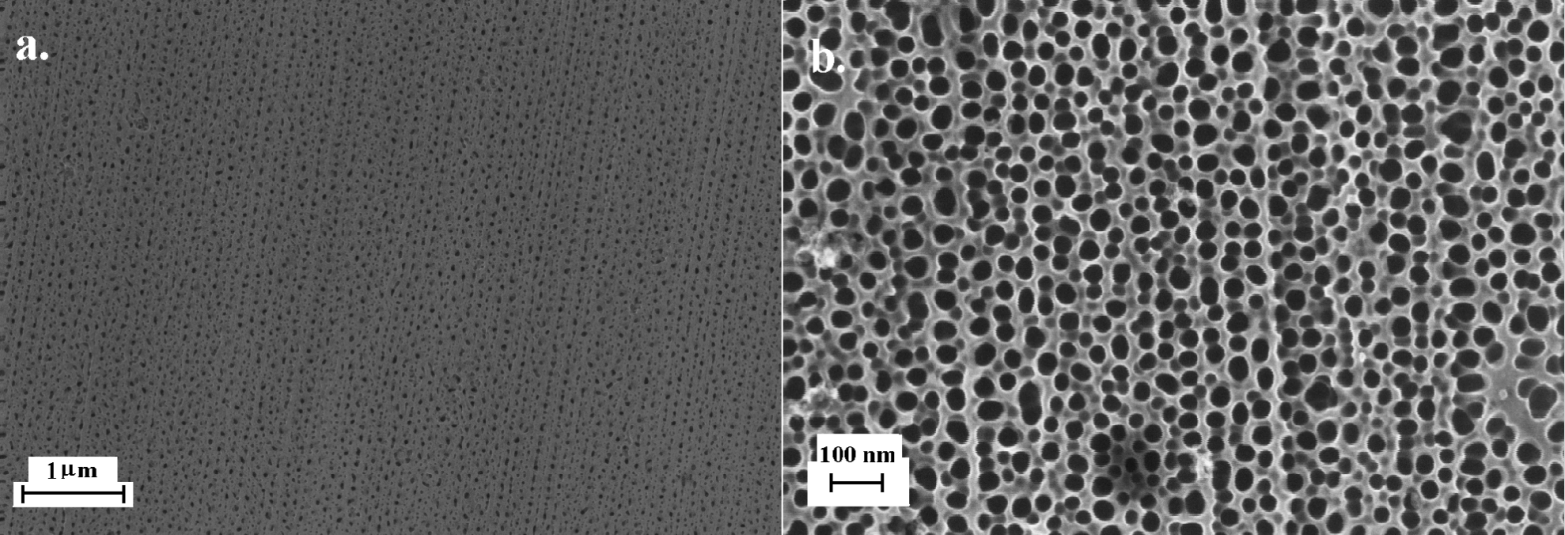
SEM images of the nanoporous alumina film anodized under constant flow conditions.

Figure 2 shows a typical SEM image of the nanoporous membrane after anodization and pore enlargement. An irregular pattern of pores with an average diameter of 30 ± 10 nm and a nearest neighbor interpore spacing of 40 ± 7 nm can be observed. The distance of the pores is about a factor of two lower compared to results obtained from standard anodization protocols reported in literature [40]. A smaller interpore spacing between nearest neighbors is expected due to the inhomogeneity of the pattern. Nevertheless, a potential drop inside the microfluidic cell might also contribute to a reduced pore separation. The striped pattern as well as the irregularity of the pores as opposed to perfect hexagonal ordering can be explained by the surface roughness of the untreated alumina samples and the single-step anodization process. An ordered pattern is usually obtained either by using imprint methods or a two-step anodization protocol [41]. At the front side, the pores were distributed over the whole surface area (0.2 mm^2^), which had been exposed to oxalic acid. Interestingly, the backside did not reveal a similar pattern but only showed nanoporous structures in a confined region of about 300 µm^2^. Such behavior is known from indented aluminum films on conducting substrates, where the indented regions exhibit advanced pore formation [15]. However, since in this investigation no inert conducting subjacent layer was present, the pore formation is expected to stop at the non-conducting alumina barrier layer after complete oxidation of the exposed aluminum film. We therefore attribute localized pore formation to the flow and diffusion profile inside the microfluidic chamber during anodization or barrier layer removal. Nevertheless, inhomogeneities in the film thickness will also affect the anodization profile and may lead to sparsely perforated regions on the backside of the membrane. A defined fluidic interface allowing precise control of the flow profile and velocity distribution could be used to study this aspect in more detail.

**Figure 3:**
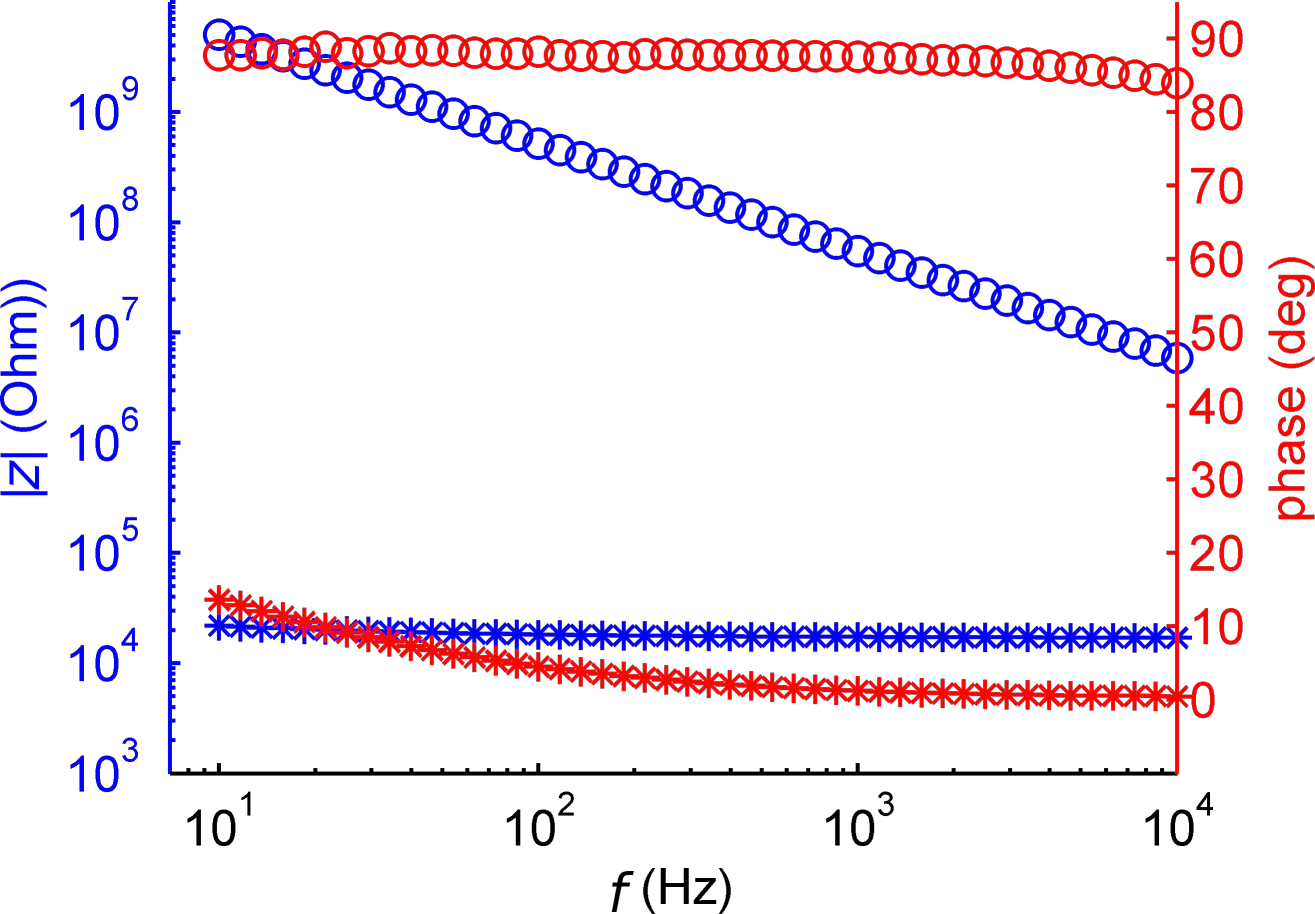
Impedance plot measured across the nanoporous membrane before (stars) and after (circles) lipid bilayer formation. Left axis: Absolute value of the impedance (blue). Right axis: Phase in degrees (red).

We investigated the formation of lipid bilayers on the nanoporous support structure inside the microfluidic cell. Figure 3 shows a plot of the impedance obtained before and after application of lipid vesicles to the front side of the silanized nanoporous membrane. The impedance measured at 10 Hz across the membrane increased by more than five orders of magnitude after vesicle application. We attribute this effect to the formation of a lipid bilayer spanning the front side of the nanoporous structure. As expected, phase changes from an ohmic to a capacitive behavior revealed the non-conducting nature of the lipid–membrane system. We chose an amino-terminated silane coating to facilitate spreading of the vesicles on the alumina surface. Vesicle rupture is probably aided via electrostatic interactions of the negative phosphate of the zwitterionic POPC-heads with the protonated amino groups from the APTES molecules immobilized on the surface [42]. In principle, one could also expect coverage of the inside pore walls, as has been observed by Bourdillon and coworkers [43–44]. However, the drastic change in impedance indicates that in our case the small pore diameter seems to favor a scenario where the nanopores are spanned by a lipid bilayer. To avoid the chemical pretreatment of the surface, an advanced deposition method of lipid bilayers on alumina as proposed by Mager et al. could be utilized [45].

Applying a simple RC equivalent circuit model to the data yields a membrane capacitance of 3.96 ± 0.1 pF. This amounts to a specific capacitance of 1.3 µF/cm^2^ for an actual active area of 300 µm^2^ in which the pores penetrate the whole substrate. This value is somewhat above the expected value of 1 µF/cm^2^ measured for a pure membrane capacitance [27]. However, discrepancies can arise because of an inaccurate determination of the size of the porous membrane patch. The impedance without the bilayer showed an almost ohmic behavior around 20 kΩ. It was partially determined by the electrolyte resistance inside the microfluidic access channels leading to the nanoporous alumina membrane.

**Figure 4:**
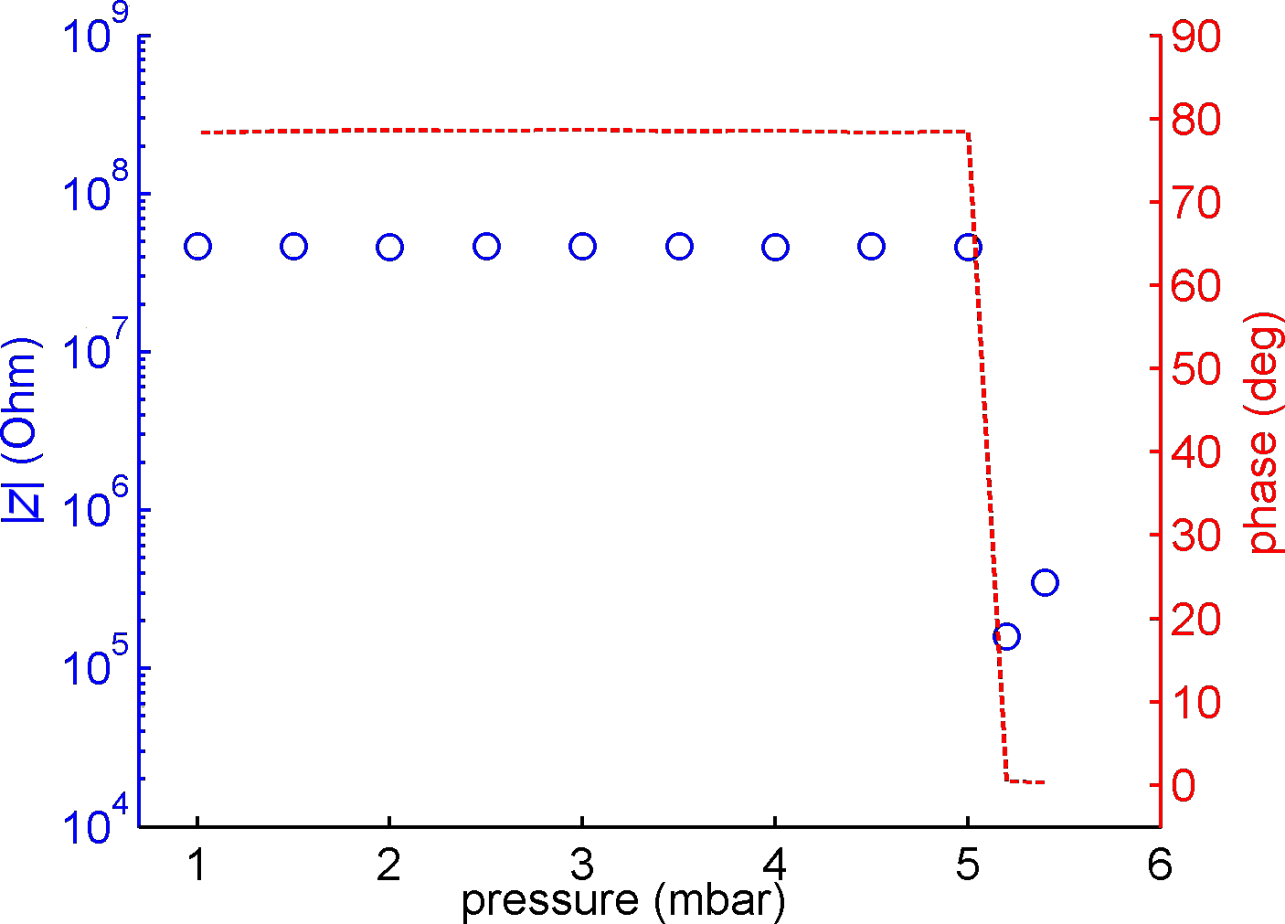
Impedance and phase measured at 1 kHz across the nanoporous membrane versus driving pressure for the microfluidic cell. A stable bilayer exhibits capacitive behavior at high impedance.

To assess the stability of the lipid bilayer under flow conditions, we measured the impedance across the membrane in dependence of the input pressure as shown in Figure 4. The bilayer could withstand an input pressure of 5.2 mbar, corresponding to a flow rate of 54 µL/min. At lower flow rates, impedance and phase exhibited a constant capacitive behavior indicating that the lipid bilayer remained intact.

**Figure 5:**
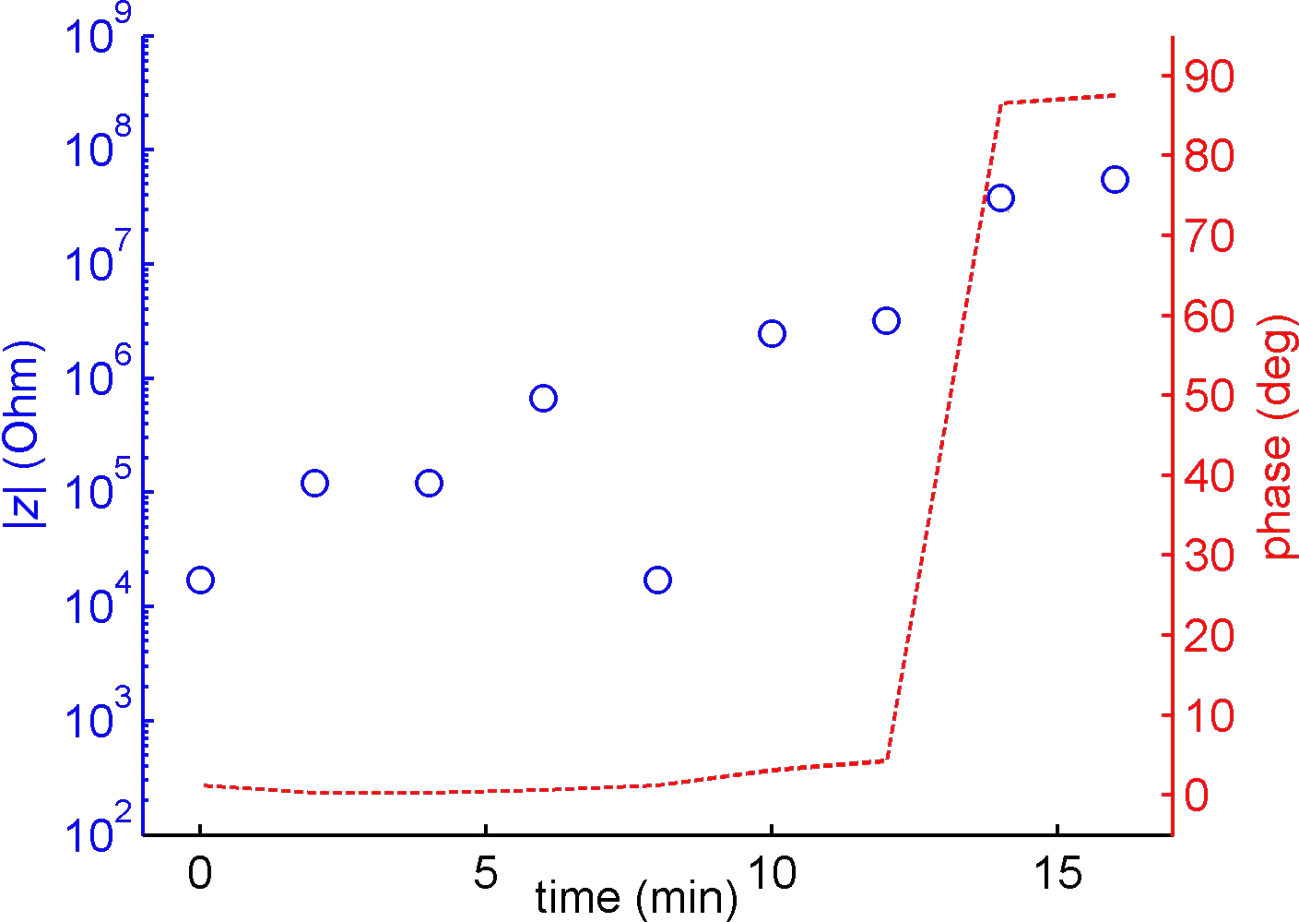
The kinetics of the lipid bilayer formation on a nanoporous alumina membrane is shown by measuring the impedance (blue) and the phase change (red) at 1 kHz across the nanoporous membrane over time.

At pressures above 5.2 mbar we observed a decrease in the impedance of several orders of magnitude and a phase shift towards a more ohmic behavior (Figure 4). The drop in impedance indicates that at least parts of the nanopore-spanning bilayer were ruptured under these flow conditions. For input pressures below 10 mbar, the rupture of the membrane was reversible. Once the pressure was lowered below 5.2 mbar we observed a steady increase of the impedance, which we attribute to the reformation of the lipid bilayer on the nanoporous substrate. The kinetics of the bilayer formation could be studied under this condition. Thus, we reversibly ruptured the membrane by raising the pressure to 10 mbar for 30 s before restoring the pressure to 0. Then we allowed the bilayer to settle and observed the change in impedance and phase at regular time intervals during the process. From Figure 5 we can see that after about 15 min the pore spanning bilayer was reestablished. However, at pressures above 10 mbar the bilayer was permanently damaged and could not be repaired by restoring the pressure. The resealing property of the membrane indicates that a residual lipid film remains on the modified alumina surface during moderate flow conditions. This film can subsequently act as a precursor to regenerate the pore spanning bilayers. The long time span of several minutes associated with this process is rather surprising. We attribute the delay to the diffusion of lipid molecules to the pore edges but further experiments will be necessary to elucidate this mechanism. The initial bilayer formation was even slower than the reformation process (~20–25 min). However, this delay can be explained by the time needed to accumulate a critical vesicle coverage required for rupture and bilayer formation [46–49].

## Conclusion

We have demonstrated a technique for locally anodizing aluminum membranes under flow conditions. Localized anodization allows the generation of stable patches of nanoporous alumina, which can be used in microfluidic experiments. Lipid membranes were grown on the nanoporous patches inside the microfluidic system and the process of membrane formation and rupture was investigated by impedance spectroscopy. We envision the use of this technique for the investigation of transmembrane protein activity under controlled flow conditions.
